# Up-regulation of SR-BI promotes progression and serves as a prognostic biomarker in clear cell renal cell carcinoma

**DOI:** 10.1186/s12885-017-3761-z

**Published:** 2018-01-22

**Authors:** Guang-hua Xu, Ning Lou, Hang-chuan Shi, Yu-chen Xu, Hai-long Ruan, Wen Xiao, Lei Liu, Xiang Li, Hai-bing Xiao, Bin Qiu, Lin Bao, Chang-fei Yuan, Ya-li Zhou, Wen-jun Hu, Ke Chen, Hong-mei Yang, Xiao-ping Zhang

**Affiliations:** 1 Department of Urology, Union Hospital, Tongji Medical College, Huazhong University of Science and Technology, No.1277, Jiefang Avenue, Wuhan, Hubei 430022 China; 2 Department of Pathogenic Biology, School of Basic Medicine, Tongji Medical College, Huazhong University of Science and Technology, No.13, Hangkong Road, Wuhan, Hubei 430030 China

**Keywords:** Scavenger receptor class B type I (SR-BI), Clear cell renal cell carcinoma (ccRCC), Progression, Prognostic biomarker

## Abstract

**Background:**

Scavenger receptor class B type I (SR-BI) has been reported to be involved in carcinogenesis of several human cancers. However, it is currently unknown whether SR-BI plays a role in clear cell renal cell carcinoma (ccRCC). Here, we aimed to evaluate a tumor promotive mechanism for SR-BI in ccRCC.

**Methods:**

The expression of SR-BI was evaluated by real-time quantitative reverse transcription polymerase chain reaction (qRT-PCR), Western blot and immunohistochemistry (IHC) in ccRCC tissues and cell lines. Lipid droplets in ccRCC tissues and normal kidney tissues were examined by Oil Red O (ORO) and hematoxylin-eosin (HE) staining. The correlation between SR-BI mRNA levels and clinicopathological features was analyzed by Pearson’s chi-square test or Fisher’s exact test. Kaplan-Meier analysis and Cox model were used to evaluate the difference in progression-free survival (PFS) associated with expression of SR-BI. Inhibition of SR-BI was conducted by using small interfering RNA (siRNA). In vitro assays were performed to assess the impact of SR-BI knockdown on cell biological behaviors. High density lipoprotein (HDL)-cholesterol content in ccRCC cells and extracellular media was also measured after transfection with siRNA.

**Results:**

The expression of SR-BI was markedly up-regulated in ccRCC tissues and tumor cell lines. ORO and HE staining revealed huge amounts of lipid droplets accumulation in ccRCC. Clinical analysis showed that over-expression of SR-BI was positively associated with tumor size, grade, distant metastasis and inversely correlated with PFS. Furthermore, SR-BI was proved to be an independent prognostic marker in ccRCC patients. The inhibition of SR-BI attenuated the tumorous behaviors of ccRCC cells, expression of metastasis and AKT pathway related proteins. The content of HDL-cholesterol was reduced in cells while increased in extracellular media after transfection with si-SR-BI.

**Conclusions:**

Our results demonstrate that SR-BI functions as an oncogene and promotes progression of ccRCC. SR-BI may serve as a potential prognostic biomarker and therapeutic target for ccRCC.

## Background

Renal cell carcinoma (RCC) represents approximately 3% in total adult malignant tumors and accounts for more than 85% of all renal malignancies arising from the kidney [1]. With a steady increase of 4.0% in the incidence and 2.3% of the mortality [2], RCC has ranked thirdly most common urological cancer. Clear cell renal cell carcinoma (ccRCC), which comprises roughly 75%~80% of all RCC cases, is the most prevalent histological subtype [3]. Due to the absence of effective diagnostic methods, along with non-specific symptoms at early stage, nearly one-third of patients present metastasis at the time of diagnosis [4]. Although surgical resection is the best strategy for localized RCC, about 25% of patients will still develop metastatic recurrence afterwards. The prognosis of RCC patients with metastasis is unfavorable, with a fact that 5-year survival rate is less than 10% [5] and the median survival time is 1.5 years [6]. Moreover, RCC is highly non-sensitive to chemotherapy and has a low response rate to cytokines therapy, with the fact that the overall survival (OS) was 8–15 months during the cytokine period [7]. With the approval of tyrosine kinase inhibitors (TKI) and mammalian target of rapamycin (mTOR) inhibitors, molecular-targeted therapy by using TKI and mTOR inhibitors has been proven to benefit the prognosis, the OS was improved up to 30 months [8], despite limited effectiveness [9]. Hence, given the grim situation, a promising biomarker with prognostic value is essential and urgently needed to put into clinical use to predict the outcome or shed light on the therapy strategy for RCC.

It has been widely accepted that metabolic disorder is an important hallmark of many cancers. Aberrant metabolism not only supports the synthesis of macromolecules, including proteins, lipids and nucleic acids, but also fuels the growth, proliferation and invasion of cancer cells [10, 11]. In addition to the abnormal glucose metabolism which is well-known as the Warburg effect, altered lipid metabolism has also been confirmed to be of great importance in the pathogenesis of cancers [12–17]. Recently, accumulating evidence reveals that RCC is a kind of metabolic disease [18–20]. Grossly, ccRCC tissue is bright and golden, and ccRCC cells are characterized by accumulation of abundant lipid droplets in cytoplasm [21]. It has been reported that ccRCC tissue contained higher levels of total cholesterol and cholesterol esters (CE) compared with normal kidney tissues [22]. Katz-Brull and Tugnoli also found that RCCs were characterized by an increase of CE by using magnetic resonance spectroscopy [23, 24]. In accordance with these results, Sundelin proved that ccRCC tissues accumulated high amounts of lipids and mainly in CE form [25]. Taking together, these results indicate a strong link between cholesterol metabolism and ccRCC.

To manifest the mechanism correlated with the accumulating of cholesterol in ccRCC, much work has been done. Schorghofer held the viewpoint that cholesterol accumulation in cancer cells derives from intracellular synthesis or uptake from outside [26]. Clayman elucidated that CE in RCC cells come from lipoprotein cholesterol in serum and the activity of low density lipoprotein receptor (LDLR) was decreased [27]. While Gebhard found that there was no increase of the HMG-CoA reductase (HMGCR) activity in RCC [22]. Rudling concluded that the mRNA levels of both HMGCR and LDLR were down-regulated in ccRCC [28]. Furthermore, it had been reported that high density lipoprotein (HDL) played the leading role in cancer progression compared with LDL [29]. Cruz [30] also clarified that HDL mediated most delivery of cholesterol from serum to malignant cells. According to the metabolic process of cholesterol, combined with the findings above, we could summarize that HDL particles contributed mostly to the intracellular accumulation of CE in ccRCC.

Scavenger receptor class B type I (SR-BI), also known as SCARB1 or CLA-1 (CD-36 and LIMPII analogous 1), a kind of membrane protein with a molecular weight of 82 kDa, acts as the major HDL receptor which mediates the selective HDL-cholesterol uptake of cells [31]. It has been proven that SR-BI functions as an oncogene and is over-expressed in a range of cancers, including nasopharyngeal carcinoma [32], prostate cancer [26, 33], breast cancer [34–37], adrenocortical cancer [38]. However, the role of SR-BI in ccRCC has not been well elucidated so far.

Therefore, the main purpose of this study was to clarify the role of SR-BI in ccRCC. We determined the expression pattern of SR-BI in ccRCC and validated its prognostic value. We also explored the biological function of SR-BI in the carcinogenesis of ccRCC by siRNA in vitro.

## Methods

### Patients and clinical tissue specimens

A total of 113 ccRCC samples and matched normal kidney tissues were collected from patients who underwent radical or partial nephrectomy at the Department of Urology, Union Hospital, Tongji Medical College, Huazhong University of Science and Technology (Wuhan, China). It was the first time for the patients to be diagnosed as ccRCC pathologically and none of them received any adjuvant treatments such as radiotherapy, chemotherapy or immunotherapy prior to surgery. All specimens were freshly snap-frozen in liquid nitrogen and stored in a deep freezer at −80 °C for RNA and protein extraction. Histological and pathological diagnosis of all specimens was confirmed by Department of Pathology, Union Hospital, Tongji Medical College, Huazhong University of Science and Technology.

### Follow up of patients

Patients’ survival condition was monitored after the operation. All the patients were followed up every 3 month in the first year, every 6 month in the second year, and then once every year thereafter. All follow-up related data or document files were acquired by phone contact or clinical examination at outpatient. The follow-up ranged from 5.06 to 90.12 months. PFS of patients was defined as the time interval from the date of first operation to the date of progression, or recurrence, or death. During the follow-up, patients whom we could not contact were recorded as censored.

### Cell culture

Human ccRCC cell lines 786-O and Caki-1 were obtained from American Type Culture Collection (ATCC, Manassas, VA, USA). HK-2, the human proximal tubule epithelial cell line, was also obtained from ATCC. All cells were cultured in Dulbecco’s modified Eagle medium (DMEM) /High Glucose (HyClone, Logan, UT, USA) supplemented with 10% fetal bovine serum (FBS, HyClone, Logan, UT, USA) and antibiotics (100 μg/μl streptomycin and 100 U/μl penicillin, HyClone, Logan, UT, USA) at 37 °C in 5% CO_2_.

### Oil Red O staining and hematoxylin-eosin staining

Oil Red O (ORO) and hematoxylin-eosin (HE) staining were conducted to compare the lipids accumulation in ccRCC and non-cancerous kidney tissues according to the manufacturer’s instructions. Classical and typical images were captured as our intention.

### RNA extraction and real-time quantitative reverse transcription PCR (qRT-PCR) analysis

Total RNA from cells and tissues was isolated with TRIzol reagent (Invitrogen, Carlsbad, CA) based on manufactures [39]. The concentration and quality of RNA were measured using Nanodrop 2000c spectrophotometer (Thermo Scientific, Waltham, MA, USA). Reverse transcription (RT) of RNA was conducted using Revertaid™ First Strand cDNA synthesis Kit (Fermentas, Vilnius, Lithuania) according to the manufacturer’s protocol. The qRT-PCR was performed with Platinum SYBR Green qPCR supermix UDG (Invitrogen, Carlsbad, CA) using synthesized primers from Genewiz (Suzhou, China). The amplification reaction conditions for SR-BI and glyceraldehyde-3-phosphate dehydrogenase (GAPDH) were 10 min at 95 °C, 45 cycles at 95 °C for 10s, then 10s at 60 °C and 15 s at 72 °C. The primer set for SR-BI was 5′-CCTATCCCCTTCTATCTCTCCG-3′ (forward), and 5′-GGATGTTGGGCATGACGATGT-3′ (reverse). GAPDH gene served as an endogenous control with primers 5′-GGTGAAGGTCGGAGTCAACGG-3′ (forward) and 5′-GAGGTCAATGAAGGGGTCATTG-3′ (reverse). The relative expression of SR-BI was analyzed by normalizing to internal control GAPDH using the 2^−ΔΔCt^ method.

### Protein extraction and western blot analysis

Total protein of tissues and cells was extracted using Radio Immunoprecipitation Assay (RIPA) buffer (Beyotime Institute of Biotechnology, Haimen, Jiangsu, China) containing protease inhibitor cocktail (Roche Molecular Biochemicals, Mannheim, Germany) and phenylmethanesulfonyl fluoride (PMSF) (Beyotime Institute of Biotechnology, Haimen, Jiangsu, China). The concentration of protein samples was determined using the Bicinchoninic acid (BCA) protein assay kit (Thermo Scientific, USA) according to the manufacturer’s procedures. Equivalent amounts of protein sample (40-60 μg) were separated by 8% dodecyl sulfate, sodium salt (SDS)-polyacrylamide gel electrophoresis (PAGE) and transferred onto polyvinylidene difluoride (PVDF) membranes (Roche, Germany). After blocking with 5% (*w*/*v*) non-fat skimmed milk at room temperature for 1 h, the membranes were washed by phosphate buffer saline (PBS)-Tween20 (PBST) for 3 times with 10 min once, and then incubated with the indicated specific primary antibody overnight at 4 °C. The primary antibodies used for western blot analysis were polyclonal rabbit anti-SR-BI (Sigma-Aldrich, SAB2700477, USA, dilution at 1:1000), polyclonal mouse anti-β-actin (Beyotime, AF0003, China, dilution at 1:3000). After washing membranes with PBST three times, the membranes were then incubated with goat anti-mouse or goat anti-rabbit secondary antibodies (Promega, Madison, WI, USA) conjugated with horseradish peroxidase (HRP) at a dilution of 1:2500 at room temperature for 2 h. After washing three times again, the stained proteins on bands were visualized and quantified using ChemiDoc-XRS+ (Bio-Rad, USA) by enhanced chemiluminescence (ECL) (Thermo Fisher Scientific Inc., Waltham, MA).

### Immunohistochemical staining

The expression pattern and localization of SR-BI protein were assessed by immunohistochemical (IHC) analysis in paraffin-embedded sections of ccRCC tissues and normal kidney tissues based on the operating steps. The primary polyclonal rabbit antibody against SR-BI (Sigma-Aldrich, SAB2700477, USA) was used at a dilution of 1:250. Representative images under the microscope were captured and recorded.

### RNA interference and transfection

Small interfering RNA (siRNA) targeting SR-BI mRNA (si-SR-BI) and scrambled negative control (si-NC) was designed and synthesized by Genepharma (Shanghai, China). The sequences of si-SR-BI were as follows: 5′-CAAGUUCGGAUUAUUUGCUTT-3′ (sense), and 5′-AGCAAAUAAUCCGAACUUGTT-3′ (antisense). Twenty-four hours before transfection, cells were seeded into a 6-well plate (Corning Costar, New York, NY, USA) at a density of (3-5) × 10^5^/ml, making sure that the confluence of cells was at about 80% the next day. Transfection of si-SR-BI or si-NC was then performed by using lipofectime2000 (Invitrogen) according to the manufacturer’s protocol, with a final concentration of 100 nmol/L. Neither antibiotics nor serum was involved during the process of transfection and the medium was replaced by new complete medium 6 h later.

### Wound closure assay

According to procedures [40], cells were seeded into a 6-well plate and cultured in complete growth medium until 80% confluences. After 48 h of siRNA transfection, a 10 μL pipette tip was used to make a vertical wound against the surface of well bottom through the cell monolayer carefully and quickly. The medium and cell debris were aspirated away carefully and cells were washed 3 times with warm PBS. Appropriate fresh culture medium was then added to the well against the wall slowly. Initial pictures of the wound at time 0 were taken under light microscope. The plate was then incubated at 37 °C in 5% CO_2_ and typical images of the wound were captured at indicated time points.

### Cell proliferation assay

The ability of cell proliferation was measured by using 3-(4, 5-dimethyl-2-thiazolyl)-2, 5-diphenyl-2-H-tetrazolium bromide (MTT) assay (Sigma, USA). Briefly, 200 μL complete DMEM/High Glucose containing 3 × 10^3^ cells was added into each well of a 96-well plate 48 h after transfection. Then, 20 μL MTT solution per well was added and the plate was incubated at 37 °C for another 4 h. At the indicated time points, the medium was removed and 150 μL dimethyl sulfoxide (DMSO) was added to dissolve the formazan crystals with shaking gently. Subsequently, absorbance values at 490 nm wavelength were measured and quantified using a microplate reader (BioTek, USA). All experiments at each time point were performed in triplicate with six replicate wells.

### Plate colony formation assay

Cells transfected with si-NC or si-SR-BI for 48 h were plated into 6-well plates at a density of 1.0 × 10^3^ cells per well and maintained in an incubator for 2 weeks with the medium replaced every 3 days. Then, cells were washed with PBS 3 times, fixed with methanol for 20 min and stained by 0.1% crystal violet for 15 min. After washing with PBS 3 times again, the images of colonies were captured and the numbers of colonies containing no less than 50 cells were counted.

### Transwell cell migration and invasion assay

The abilities of cell migration and invasion in vitro were evaluated by performing transwell assays. As described before [41], A 24-well transwell chamber with 8 μm pore size (Corning Costar, New York, NY, USA) was used for this experiment. Briefly, cells were seeded into 6-well plates and transfected with si-NC or si-SR-BI. After transfection for 24 h, cells were harvested, centrifuged and resuspended with serum-free culture medium. Cells were then counted under microscope to get the concentration. Subsequently, cells contained in 100 μL culture medium with no serum were loaded into upper chamber at a pre-determined density (3.0 × 10^4^/well for 786-O and 5.0 × 10^4^/well for Caki-1). While in the lower insert, 500 μL complete medium with 10% FBS was added as a kind of chemo-attractant. It should be noted that the upper chamber used for invasion assay was pre-coated appropriate amount matrigel (BD Biosciences) at a dilution of 1:8 and dried in a cell incubator for 5 h prior to cell seeding. Twenty-four hours post-incubation at 37 °C in 5% CO_2_, cells remained on the upper surface of the membrane were removed gently, whereas those migrated the filter membrane and attached to the lower side of it were fixed by methanol and stained with 0.1% crystal violet after washing with PBS. Photographs were taken and cells number was counted microscopically.

### Determination of HDL-cholesterol content

To explore whether HDL-cholesterol content was changed after down-regulation of SR-BI, we tested and quantified the content in ccRCC cells and extracellular media after transfection with siRNA in 786-O and Caki-1 cells. HDL-cholesterol content was determined by using the HDL-cholesterol kit (Nanjing Jiancheng bioengineering Institute, A112-1, Nanjing, China) following manufacturer’s instructions. Transfected cells were also stained with ORO to assess the changes.

### Statistical analysis

All statistical analyses were performed by SPSS16.0 software (SPSS, Inc., Chicago, IL, USA) or GraphPad Prism version5.01 (GraphPad Software, Inc., CA, USA). All continuous results were expressed as means ± standard deviation (SD). A *P*-value <0.05 was considered statistically significant.

## Results

### Enhanced SR-BI expression in ccRCC tissues and cell lines

**Fig. 1:**
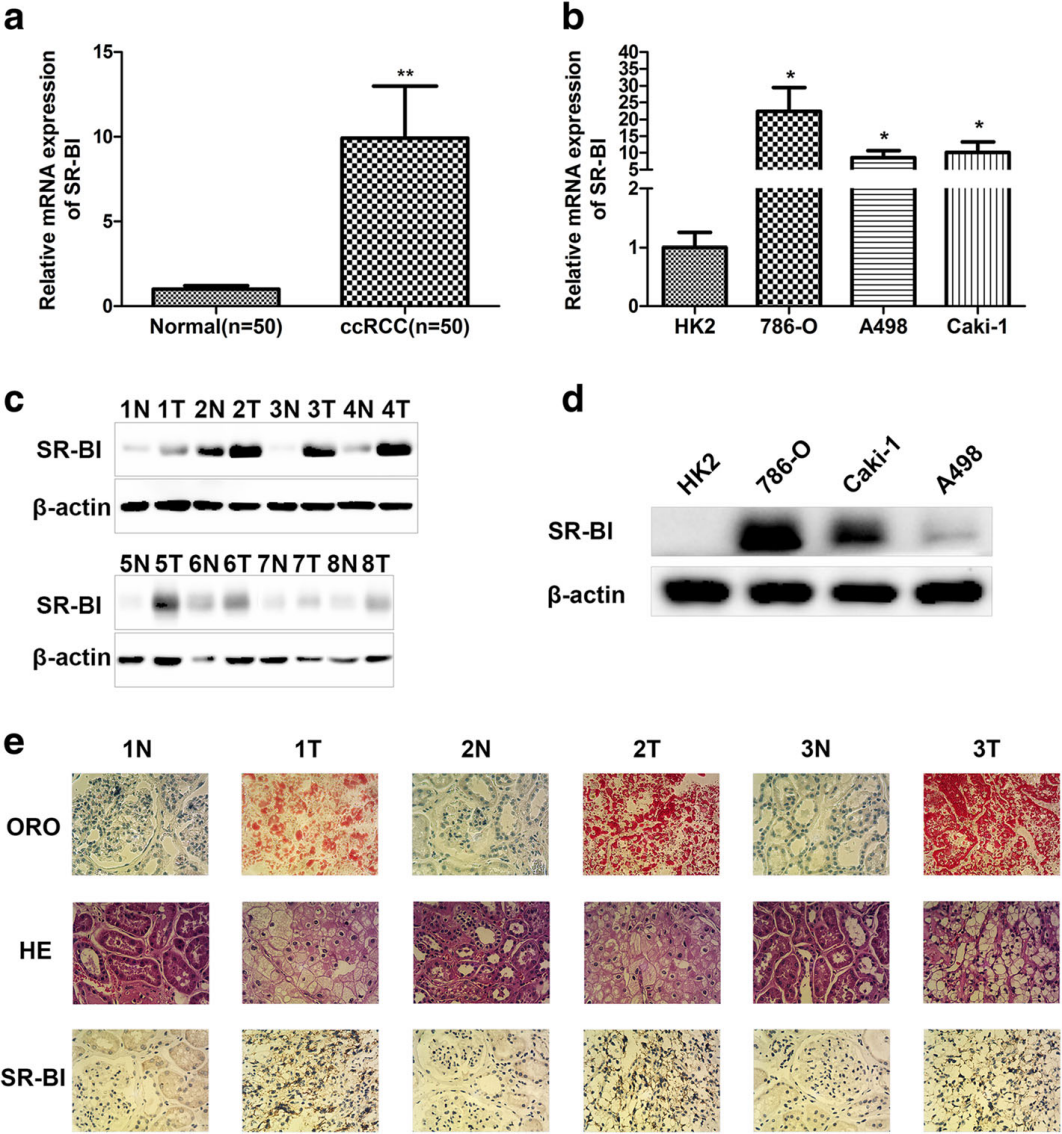
Overexpression of SR-BI in ccRCC. Relative mRNA expression of SR-BI in normal kidney and ccRCC tissues (**a**), normal kidney cell line and ccRCC cell lines (**b**) by qRT-PCR. Protein expression of SR-BI in normal kidney and ccRCC tissues (**c**), normal kidney cell line and ccRCC cell lines (**d**) by western blot. Representative images of ORO staining, HE staining and IHC analysis of SR-BI protein in normal kidney and ccRCC tissues (**e**) (original magnification ×400). *P < 0.05, **P < 0.01

It has been reported that SR-BI played vital roles in several human cancers. However, we know little about SR-BI in ccRCC. Hence, to explore the role of SR-BI in ccRCC, we examined SR-BI expression in ccRCC tissues and cell lines by using qRT-PCR and western blot. As shown in Fig. 1a-c, the expression of SR-BI was significantly increased in ccRCC tissues compared with normal matched tissues. Furthermore, a similar result was observed in tumor cell lines. As shown in Fig. 1b-d, the expression of SR-BI was significantly increased in 786-O and Caki-1 cells compared with HK-2 cells. IHC staining also showed that protein level of SR-BI was much higher in cancerous tissues (Fig. 1e).

### Increased accumulation of lipid droplets in ccRCC tissues

To verify that there are many lipid droplets in ccRCC tissues, ORO and HE staining was performed. By using the light microscope, we found that lipid droplets were widely stained red in ccRCC tissues, whereas no obvious difference was observed in paired normal kidney samples by ORO staining. HE staining also showed much higher content of lipid droplets in cancerous tissues (Fig. 1e).

### Associations between SR-BI mRNA expression and clinicalpathological features of ccRCC

To explore the clinical significance of SR-BI expression in ccRCC, correlation of SR-BI mRNA expression with clinical-pathological data was analyzed. SR-BI expression was used to assign patients to the high (upper 50th percentile) or low (lower 50th percentile) expression groups. As shown in Table 1, the relationship between SR-BI mRNA expression level and the clinical-pathological variables was analyzed by Chi-square test or Fisher’s exact test. The high expression of SR-BI in ccRCC was critically associated with tumor size (*P* = 0.019), grade (*P* = 0.040), distant metastasis (*P* = 0.006).

**Table 1:** Correlation between SR-BI mRNA expression and clinicopathological parameters of ccRCC patients

		SR-BI mRNA expression		
Features	Number	Low (*n* = 56)	High (*n* = 57)	*P*
Gender				0.146
Male	67	37	30	
Female	46	19	27	
Age (years)				0.290
≤ 60	74	34	40	
> 60	39	22	17	
Tumor size (cm)				**0.019**
≤ 4.0	33	22	11	
> 4.0	80	34	46	
Furman grade				**0.040**
G1 + G2	75	32	43	
G3 + G4	38	24	14	
Tumor stage				0.222
T1 + T2	83	44	39	
T3 + T4	30	12	18	
N metastasis				0.298
Negative	97	50	47	
Positive	16	6	10	
M metastasis				0.006*
M0	105	56	49	
M1	8	0	8	
TNM stage				0.144
I + II	74	40	34	
III + IV	39	15	23	
Venous invasion				0.283*
Yes	8	2	6	
No	105	54	51	
Location side				0.512
Left	58	27	31	
Right	55	29	26	

*Fisher’s exact test

### Prognostic value of SR-BI mRNA expression in ccRCC

**Fig. 2:**
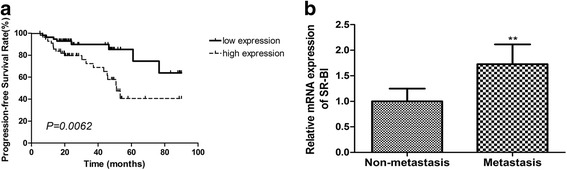
Prognostic value of SR-BI expression in ccRCC patients. **a** Kaplan–Meier analysis indicated that patients with tumors that expressed high levels of SR-BI had a shorter PFS. **b** Mann–Whitney test showed that patients with tumors that expressed high SR-BI levels had a stronger potential of distant metastasis. **P < 0.01

To further clarify the clinical value of SR-BI mRNA expression in ccRCC patients, Kaplan-Meier analysis and log-rank test was conducted to determine whether the PFS was associated with the expression of SR-BI in tumors. As shown in Fig. 2a, the patients with tumors that expressed high levels of SR-BI had a shorter PFS survival (*P* = 0.0062). In addition, we also detected the mRNA expression of SR-BI in ccRCC cancerous tissues with distant metastasis. As shown in Fig. 2b, the expression of SR-BI in patients with distant metastasis was significantly higher than those without metastasis (*P* = 0.0019). To further confirm whether SR-BI mRNA level was an independent prognostic factor of clinical outcome, Cox proportional hazards regression models were performed. By using univariate analysis (Table 2), the gender (*P* = 0.029), tumor size (*P* = 0.004), lymph node metastasis (*P* < 0.001), distant metastasis (*P* = 0.001), TNM stage (*P* < 0.001) and SR-BI mRNA level (*P* = 0.009) were significantly related to the PFS of ccRCC patients, suggesting SR-BI serves as a prognostic factor. However, multivariate analysis (Table 2) indicated that only increased SR-BI mRNA expression (*P* = 0.021), gender (*P* < 0.001) and tumor size (*P* = 0.002) were independent prognostic factors.

**Table 2:** Cox regression analysis of PFS rate in 113 ccRCC patients

Variables	Univariate analysis			Multivariate analysis		
	HR	95%CI	*P* value	HR	95%CI	*P* value
Gender	0.384	0.163–0.908	**0.029**	0.131	0.046–0.374	**0.000**
Age	1.144	0.526–2.489	0.735			
Tumor size	8.742	2.036–37.546	**0.004**	11.696	2.433–56.216	**0.002**
Furman grade	1.704	0.800–3.628	0.167			
T stage	1.610	0.700–3.703	0.263			
N metastasis	5.593	2.409–12.984	**0.000**	2.786	0.904–8.580	0.074
M metastasis	5.730	2.107–15.583	**0.001**	2.221	0.714–6.909	0.168
TNM stage	4.363	2.014–9.451	**0.000**	1.895	0.654–5.494	0.239
Venous invasion	1.605	0.483–5.338	0.440			
Location side	1.022	0.487–2.147	0.954			
SR-BI expression	3.014	1.317–6.898	**0.009**	2.926	1.175–7.285	**0.021**

*HR* hazard ratio, *CI* confidence interval

### Knockdown of SR-BI suppresses ccRCC cells proliferation and plate colony formation

**Fig. 3:**
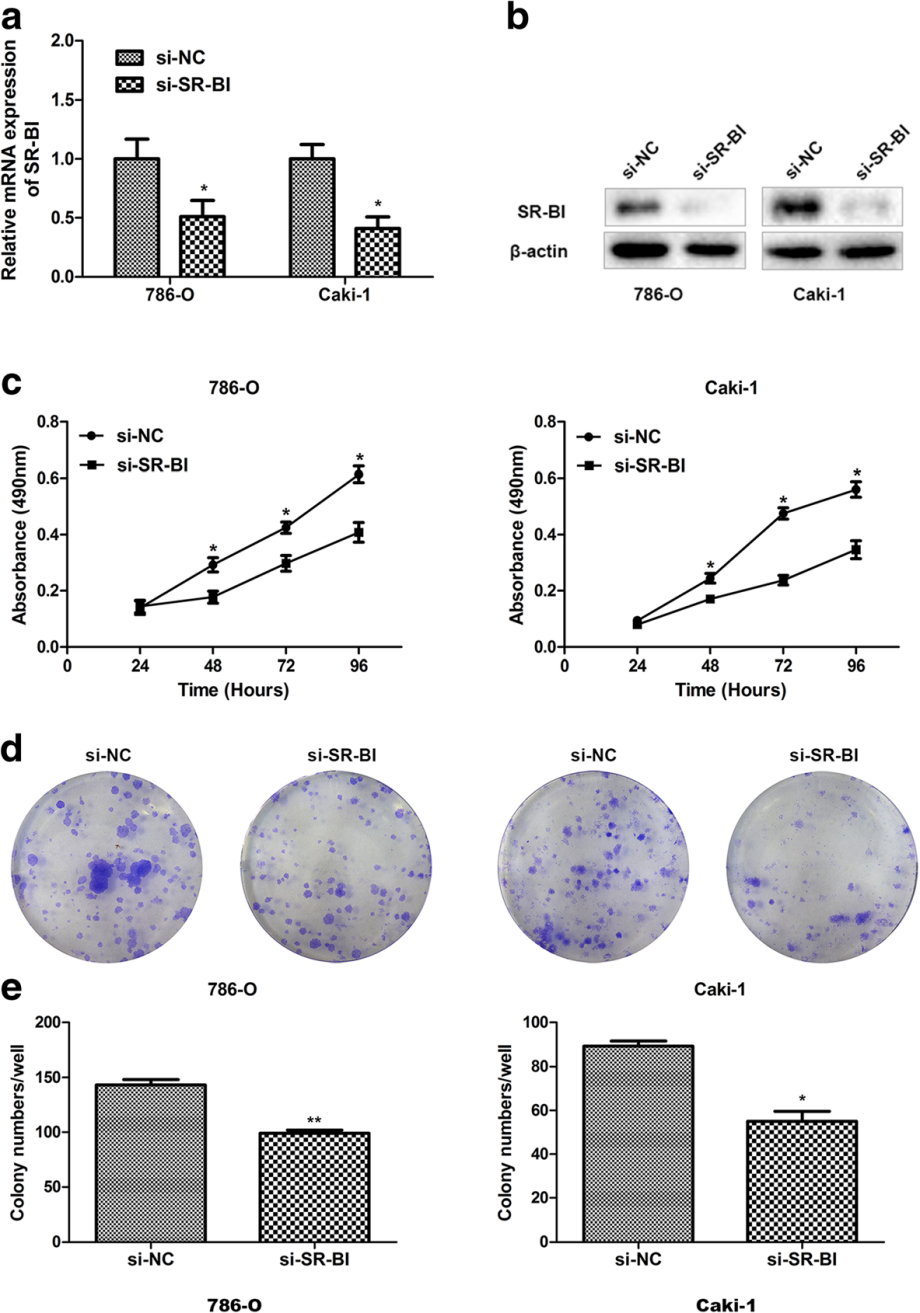
Knockdown of SR-BI inhibited the growth of ccRCC cells. Expression of SR-BI mRNA (**a**) and protein (**b**) was effectively inhibited by specific siRNA in ccRCC cell lines. MTT assays showed that proliferative ability of ccRCC cells transfected with si-SR-BI significantly decreased compared with control cells (**c**). Plate colony formation assays exhibited that ccRCC cells transfected with si-SR-BI formed fewer colonies than control cells (**d-e**). *P < 0.05, **P < 0.01

To exploit the biological function of SR-BI in ccRCC carcinogenesis and progression, we used siRNA to knockdown endogenous SR-BI expression in vitro. As shown in Fig. 3a-b, SR-BI expression was effectively inhibited by siRNA in ccRCC cell lines. MTT assay was then conducted to assess the impact of SR-BI on cell proliferation. The results showed that knockdown of SR-BI could significantly decrease the proliferative ability of ccRCC cells (Fig. 3c). In consistence with the results, ccRCC cells transfected with si-SR-BI formed fewer colonies than those transfected with si-NC (Fig. 3d-e).

### Knockdown of SR-BI impairs ccRCC cells invasion and migration in vitro

**Fig. 4:**
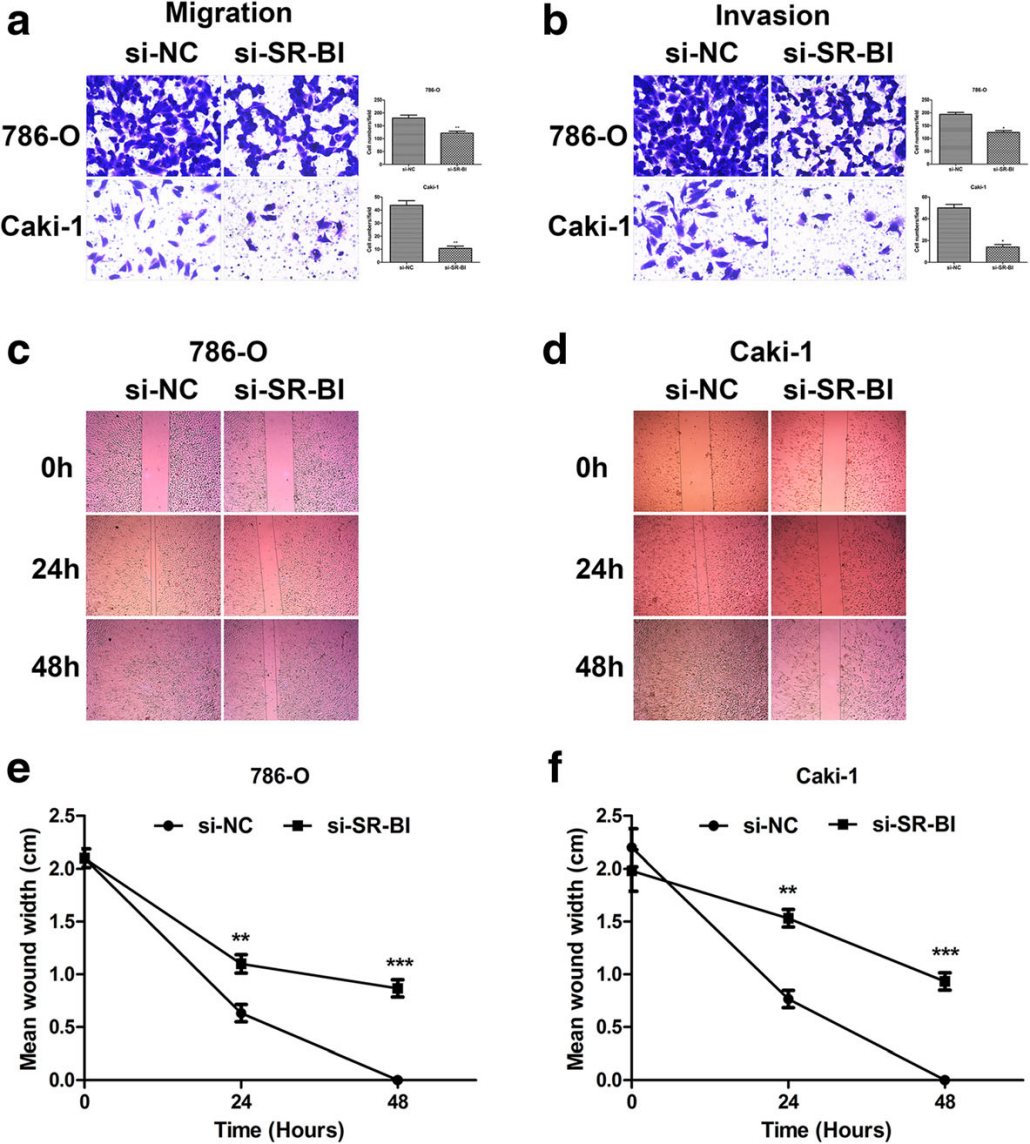
Knockdown of SR-BI attenuated migration (**a**) and invasion (**b**) of ccRCC cells. Wound closure assays showed that 786-O (**c, e**) and Caki-1 (**d**, **f**) cells transfected with si-SR-BI healed the gaps much slower than control cells at the indicated time points. *P < 0.05, **P < 0.01, ***P < 0.001

The capacity of cell invasion and migration means much in cancer development, progression and metastasis. Next, we explored whether depletion of endogenous SR-BI could attenuate the invasion and migration of ccRCC cells in vitro. Transwell invasion and wound healing assays were performed to verify the hypothesis. As shown in Fig. 4a-b, ccRcc cells treated with si-SR-BI reduced the potential of migration and invasion dramatically compared with the cells transfected with si-NC. Meanwhile, the number of migrated cells treated with si-SR-BI was much fewer. Accompanied by the results, wound healing assay proved that SR-BI knockdown closed the gap much slower (Fig. 4c-f).

### Knockdown of SR-BI inhibited the up-take of HDL-cholesterol

**Fig. 5:**
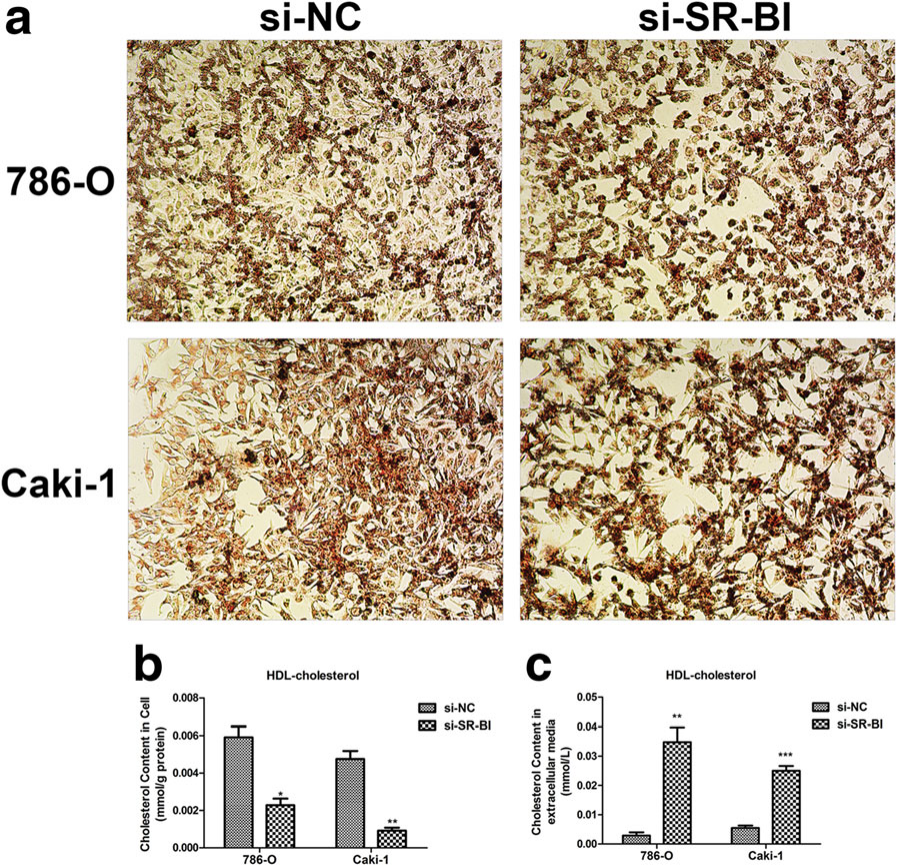
Knockdown of SR-BI inhibited the up-take of HDL-cholesterol. Representative images of ORO staining for ccRCC cells after transfection with siRNA (**a**). The cholesterol content in ccRCC cells transfected with specific si-SR-BI was much less than control cells (**b**). The cholesterol content in extracellular media was significantly higher after cells transfection with si-SR-BI (**c**). *P < 0.05, **P < 0.01, ***P < 0.001

As shown in Fig. 5a, ccRCC cells transfected with siRNA were stained by ORO, and we found that knockdown of SR-BI inhibited the proliferation of cancer cells. More importantly, cells transfected with si-NC showed much fresher red color than cells treated with si-SR-BI, which meant that there was much lower HDL-cholesterol content in cells treated with si-SR-BI, when HDL receptor was knocked down. The quantification test also showed that there was much less HDL-cholesterol in cells (*P* = 0.0147 for 786-O, *P* = 0.0015 for Caki-1) while much more in extracellular media (*P* = 0.0050 for 786-O, *P* = 0.0007 for Caki-1) with down-regulation of SR-BI (Fig. 5b-c).

### Knockdown of SR-BI induced the reduction of AKT, p-AKT and MMP-2/9

**Fig. 6:**
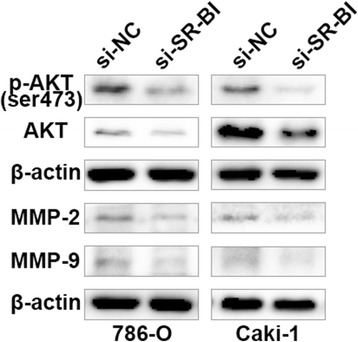
Reduction of SR-BI expression induced by si-SR-BI suppressed the AKT pathway and MMP-2/9 expression

It has been widely recognized that PI3K/AKT pathway is tightly correlated with cell metabolisms [42, 43]. More persuasively, the concept was verified in prostate cancer [16]. Recently, It is well known that matrix metalloproteinases (MMP) especially MMP-2/9 played vital roles in cancer progression [44, 45]. Hence, we confirmed whether knockdown of SR-BI would influence the expression of AKT, p-AKT and MMP-2/9. As shown in Fig. 6, the protein expression of AKT, p-AKT and MMP-2/9 were all dramatically decreased when ccRCC cells were transfected by si-SR-BI (Fig. 6).

## Discussion

RCC, especially ccRCC, has always been regarded as genetic disorder disease. As the most predominant subtype of RCC, ccRCC is featured by loss of heterozygosity (LOH) on chromosome 3p or direct deletion of chromosome 3p [46, 47]. Additionally, mutation or silencing of tumor suppressive genes such as *VHL* and *PTEN*, accompanied by the activation of oncogenes sometimes, contributes to the carcinogenesis of ccRCC. However, within recent decades, mushroomed researches revealed the importance of cell metabolism in tumorigenesis. Energy metabolism of cells derived from lipids, glucose or other nutrients has emerged a vital hallmark of tumors [48].

To better understand the pathogenesis and clinical outcomes of ccRCC, to supply molecular-targeted therapy, a sort of precise medicine to patients, then, as the basis and core of the novel therapy mode, biomarkers were focused on by numerous studies. Much has been done to explore biomarkers, aiming to uncover their roles in ccRCC, just like the role of prostate specific antigen (PSA) in diagnosis and prognosis of prostate cancer. Despite intensive efforts, the clinical value of biomarkers in ccRCC has not been elucidated fully and clearly, especially those involved in lipids metabolism.

SR-BI, a membrane protein which was well-known as a mediator for hepatitis C virus entry into hepatic cells or lipids transport in normal cells once [49–52], has been identified to participate actively in carcinogenesis recently. Unfortunately, the mechanism underlying the up-regulation of SR-BI in ccRCC maintains indistinct and misty.

In the present study, based on the histopathological appearance of ccRCC tissues, we demonstrated the accumulation of lipid droplets in ccRCC tissues and hypothesized the clinical significance of lipids excess to cancer cells subsequently. Furthermore, we ascertained the role of SR-BI, the key molecule involved, in the development and progression of ccRCC.

We started by staining the ccRCC tissues using ORO and HE dyestuff to assess the lipids content. Consistent with the previous studies [25, 53], we also found that much more lipids accumulated in cancerous tissues than in normal kidney tissues, despite different molecules that contributed to the results. Then, we examined SR-BI expression in ccRCC tissues and cells. We confirmed that the expression of SR-BI was up-regulated in cancerous tissues and cell lines compared with their normal counterparts. These results were similar with those in other cancer types [26, 32–38].

Next, we analyzed the correlation between SR-BI mRNA expression and clinical factors, and the result showed that overexpression of SR-BI was significantly related to tumor size, grade and M metastasis. The result indicated that high expression of SR-BI may participate in the aggressiveness and metastasis of ccRCC, according to its expression pattern in other cancers. In addition, we also speculated that tumor with bigger size or higher grade had the higher possibility of progression. Similarly, Schorghofer also reported that high SR-BI expression was significantly associated with Gleason score, an important clinical feature in prostate cancer [26].

Naturally, we subsequently pursued to find out the prognostic value of SR-BI expression in ccRCC. As a retrospective study, we obtained the PFS time of patients by follow-up, and we identified that high SR-BI mRNA expression was associated with shorter PFS. Univariate and multivariate analysis showed that high SR-BI mRNA level was an independent prognostic biomarker for PFS. Then, we concluded that high expression of SR-BI meant an unfavorable outcome in ccRCC. Schorghofer held similar viewpoints with a fact that lower SR-BI expression was related to longer disease-free survival time in prostate cancer [26].

After focusing on SR-BI at tissue level, we explored to elucidate the biological function in tumor cells. RNA interfering was applied to inhibit expression of SR-BI in vitro. After transfection of siRNA in ccRCC cell lines, we examined that expression of SR-BI was inhibited effectively. Knockdown of SR-BI dramatically decreased the viability and colony formation ability of ccRCC cells. Similar results were observed in previous studies that depletion of SR-BI either by RNA interfering or gene mutation critically reduce the growth of cancer cells [33, 35, 36]. In addition, we confirmed that depletion of SR-BI significantly reduced cell invasion and migration. Similarly, Danilo also demonstrated that knockdown of SR-BI vitally inhibited the invasion of breast cancer cells despite there was no difference in cell migration [35].

As the receptor of HDL-cholesterol, SR-BI downregulation inhibited the up-take of HDL-cholesterol and proliferation of ccRCC cells. The results were in accordance with conclusions from Danilo in breast cancer research [35]. The data suggested that HDL-cholesterol may participate in the proliferation of ccRCC cells and act as a kind of energy resource for progression.

However, the mechanism(s) by which SR-BI augments the invasion ability of ccRCC has not been well elucidated. Cao et al. reported that the PI3K/Akt signaling pathway played vital roles in CAL-1 (SR-BI) mediated breast cancer progression, mutant form of SR-BI impairs the proliferation of MCF-7 cells and the effect was induced by inactivation of PI3K/Akt pathway [36]. Danilo revealed that activation of Akt cascades was induced by SR-BI and knockdown of SR-BI impair the effects [35]. Fruhwurth also shed out direct link between mTOR pathway and SR-BI expression, inhibition of mTOR pathway decreased SR-BI expression and nitric oxide (NO) synthesis in human umbilical vein endothelial cells (HUVECs) and human coronary artery endothelial cells (HCAECs) [54]. While NO stimulated the vascular endothelial growth factor-C (VEGF-C) expression and promoted lymph node metastasis in breast cancer [55].

In our studies, we evaluated that AKT and p-AKT protein expression in ccRCC cell lines treated with si-SR-BI were decreased dramatically. Besides, down-expression of MMP-2/-9 was also observed, which was similar to the results from Ji [56]. In the future, molecule mechanism(s) of SR-BI involved in ccRCC should be deeperly explored.

## Conclusions

Our results confirmed that SR-BI was up-regulated in ccRCC tissues and cell lines. High SR-BI expression correlated tightly with aggressive features of ccRCC and predicted an unfavorable clinical outcome. We further identified that inhibition of SR-BI in ccRCC cell lines in vitro using specific siRNA successfully damaged the growth, colony formation, migration and invasion, as well as the up-take of HDL-cholesterol. Taking together, our data suggested that SR-BI overexpression contributed to progression of ccRCC and might serve as an independent prognostic biomarker.

## Abbreviations

AUC: Area under the curve
BCA: Bicinchoninic acid
ccRCC: Clear cell renal cell carcinoma
CE: Cholesterol esters
CI: Confidence interval
DMEM: Dulbecco’s modified Eagle medium
DMSO: Dimethyl sulfoxide
GAPDH: Glyceraldehyde-3-phosphate dehydrogenase
HDL: High density lipoprotein
HE: Hematoxylin-eosin
HMGCR: HMG-CoA reductase
HRP: Horseradish peroxidase
IHC: Immunohistochemistry
LDLR: Low density lipoprotein receptor
LOH: Loss of heterozygosity
MMP: Matrix metalloproteinases
mTOR: Mammalian target of rapamycin
MTT: 3-(4, 5-dimethyl-2-thiazolyl)-2, 5-diphenyl-2-H-tetrazolium bromide
NC: Negative control
ORO: Oil Red O
PAGE: Polyacrylamide gel electrophoresis
PBS: Phosphate buffer saline
PMSF: Phenylmethanesulfonyl fluoride
PSA: Prostate specific antigen
PVDF: Polyvinylidene difluoride
qRT-PCR: Real-time quantitative reverse transcription polymerase chain reaction
RCC: Renal cell carcinoma
RIPA: Radio Immunoprecipitation Assay
ROC: Receiver operating characteristic
SDS: Dodecyl sulfate, sodium salt
siRNA: Small interfering RNA
SR-BI: Scavenger receptor class B type I
